# MicroRNAs as the critical regulators of tyrosine kinase inhibitors resistance in lung tumor cells

**DOI:** 10.1186/s12964-022-00840-4

**Published:** 2022-03-09

**Authors:** Amirhosein Maharati, Amir Sadra Zanguei, Ghazaleh Khalili-Tanha, Meysam Moghbeli

**Affiliations:** 1 Student Research Committee, Faculty of Medicine, Mashhad University of Medical Sciences, Mashhad, Iran; 2 Department of Medical Genetics and Molecular Medicine, School of Medicine, Mashhad University of Medical Sciences, Mashhad, Iran

**Keywords:** Non-small cell lung cancer (NSCLC), Tyrosine kinase inhibitor (TKI), Resistance, MicroRNA (miRNA), Diagnosis, Prognosis

## Abstract

Lung cancer is the second most common and the leading cause of cancer related deaths globally. Tyrosine Kinase Inhibitors (TKIs) are among the common therapeutic strategies in lung cancer patients, however the treatment process fails in a wide range of patients due to TKIs resistance. Given that the use of anti-cancer drugs can always have side effects on normal tissues, predicting the TKI responses can provide an efficient therapeutic strategy. Therefore, it is required to clarify the molecular mechanisms of TKIs resistance in lung cancer patients. MicroRNAs (miRNAs) are involved in regulation of various pathophysiological cellular processes. In the present review, we discussed the miRNAs that have been associated with TKIs responses in lung cancer. MiRNAs mainly exert their role on TKIs response through regulation of Tyrosine Kinase Receptors (TKRs) and down-stream signaling pathways. This review paves the way for introducing a panel of miRNAs for the prediction of TKIs responses in lung cancer patients.

**Video Abstract**

**Video Abstract**

## Background

**Fig. 1:**
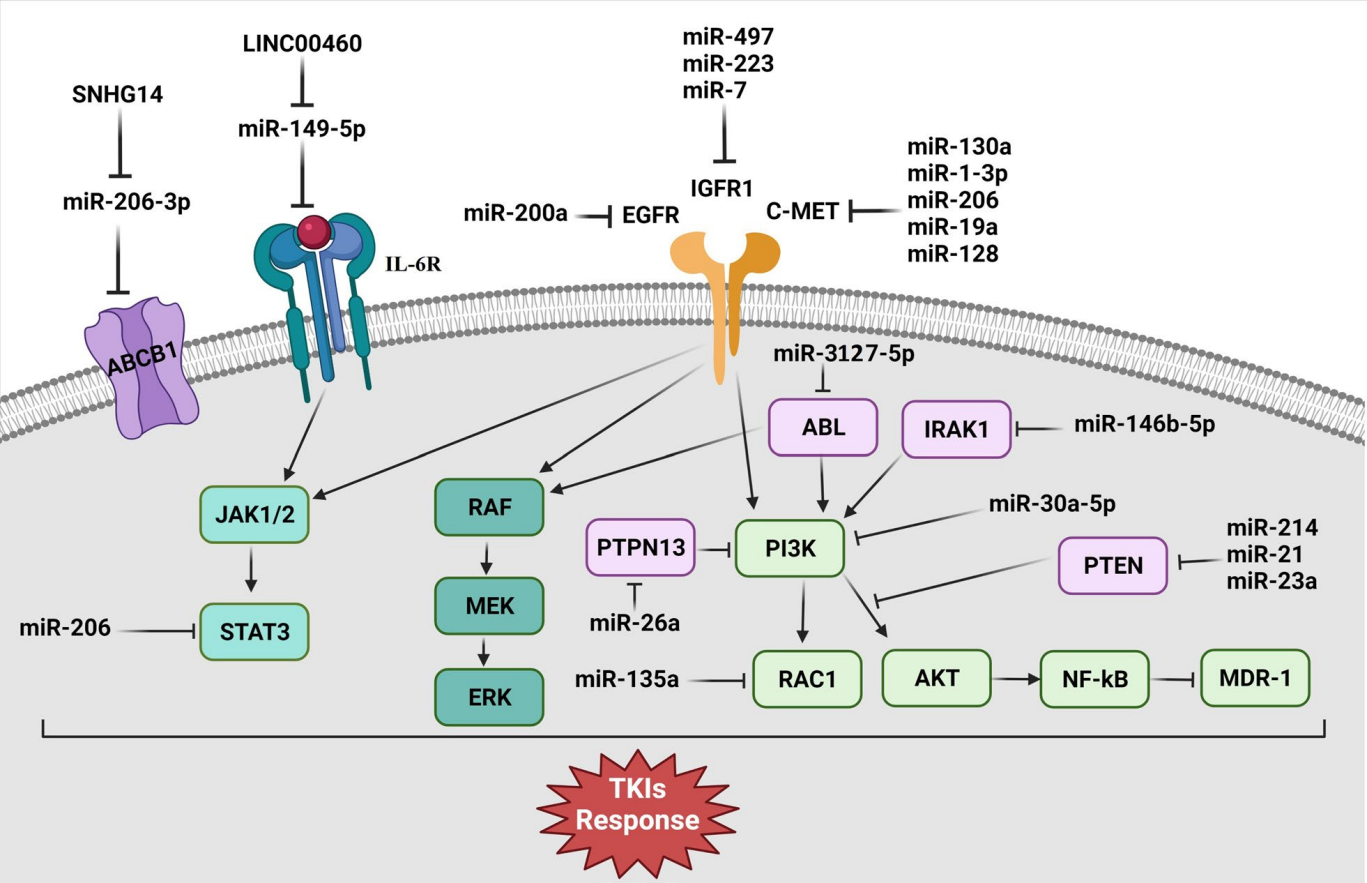
Molecular mechanisms of microRNAs involved in regulation of TKIs responses in lung tumor cells. All of the microRNAs that targeted the RTKs were involved in increased TKIs sensitivity in lung tumor cells. MiR-214, miR-21, and miR-23a promoted TKIs resistance through PTEN targeting. SNHG14 and LINC0060 also increased TKIs resistance by miR-206-3p and miR-149-5p targeting and following ABCB1 and IL-6 up regulations in lung tumor cells. MiR-3127-5p and miR-146b-5p were also involved in increased TKIs sensitivity through ABL and IRAK1 targeting, respectively. (Created with BioRender.com)

Lung cancer is the leading cause of cancer-related mortality and the third most common cancer worldwide [1]. Lung cancers are classified into two broad categories based on histopathological features: Non-Small-Cell Lung Cancer (NSCLC) and Small-Cell Lung Cancer (SCLC). NSCLC accounts for almost 85% of newly diagnosed lung cancer cases and has three main subclasses: adenocarcinoma, squamous-cell carcinoma, and large-cell carcinoma [2]. While tremendous progress has been achieved in the last decade, the prognosis for lung cancer remains poor, with just 19% of patients survive for longer than five years. A substantial proportion of NSCLC patients have genetic alterations in Epidermal Growth Factor Receptor (EGFR) that activate it constitutively [3–6]. Tyrosine kinases are categorized into the trans-membrane Receptor Tyrosine Kinases (RTKs) and cytoplasmic Non-Receptor Tyrosine Kinases (NRTKs) [7]. RTKs are involved in both extracellular and intracellular signaling pathways. They often serve as binding sites for cytoplasmic molecules that activate downstream pathways. RTK ligand binding triggers receptor dimerization and auto-phosphorylation that results in activation of downstream signaling molecules involved in cell proliferation and tumor progression [8]. EGFR is a transmembrane glycoprotein belonging to the Receptor Tyrosine Kinases (RTKs) family that activates Mitogen-Activated Protein Kinase (MAPK) and Phosphatidylinositol 3-Kinase (PI3K)/protein kinase B (AKT), and Janus Kinase (JAK)/Signal Transducer and Activator of Transcription (STAT) signaling pathways to regulate cell proliferation and angiogenesis [9–11]. EGFR deregulations are found in various cancers [12, 13]. NRTKs usually have interaction with transmembrane receptors and transduce extracellular signals. They have also a critical role in regulation of cell proliferation, apoptosis, and immune response through PI3K/AKT and MAPK signaling pathways [14, 15]. Targeting the EGFR is authorized as a first-line treatment option for NSCLC patients with an activating *EGFR* mutation and a second-line in patients with advanced NSCLC [16–19]. EGFR-Tyrosine Kinase Inhibitors (TKIs) inhibit EGFR phosphorylation and thus interfere with MEK-ERK, PI3K-AKT, and JAK-STAT activation. For locally developed or metastatic NSCLC with mutant EGFR, oral administration of EGFR-TKIs such as Gefitinib, Erlotinib, Afatinib, and Osimertinib are the conventional treatment options [20, 21]. Nevertheless, specific NSCLC patients who carry EGFR-TKI-sensitizing mutations do not respond to EGFR-TKIs. Resistance to EGFR-TKIs develops in approximately one year that severely reduces the long-term efficiency [22, 23]. MicroRNAs (miRNAs) are non-coding RNAs that regulate gene expression through translational repression and mRNA cleavage [24]. MiRNAs functions as oncogenes or tumor suppressor genes in regulating cell proliferation and apoptosis [25, 26]. They regulate cancer cell susceptibility to chemotherapy and prevent tumor cell motility and invasion [27–30]. They can also down-regulate the EGFR signaling transduction while restoring Gefitinib cytotoxicity in NSCLC cells [31]. Although, TKIs are effective therapeutic modalities in the targeted therapy of various cancers, they cause various adverse effects on skin and hair, anemia, hypothyroidism, and diarrhea [32]. Therefore, it is required to detect the lung cancer patients who are resistant toward the TKIs to manage the therapeutic methods and reduce side effects. Since, the miRNAs are stable in body fluids; they can be used as the non-invasive diagnostic and prognostic markers [33, 34]. Therefore, in the present review we have discussed the miRNAs involved in regulation of TKIs response in lung cancer (Fig. 1) (Table 1).

**Table 1:** All of the microRNAs associated with TKIs response in lung tumor cells

Gene	Samples	Results	Animal study	References
*Enhancing sensitivity to TKIs*				
miR-1	• 21 tumor tissues • 21 resistant tumor tissue • PC9 and H1975 cell lines	Inhibited monocytes and lymphocyte motility by cytokines down regulations and reduced intra-tumoral CD8-positive T cells	–	Kawana [60]
miR-1-3p	• HCC827 and PC9 cell lines • Xenograft model	Overcome HGF-induced Gefitinib resistance in EGFR mutant lung cancer cells by AKT/ERK inhibition	MiR-1-3p reversed HGF-induced resistance to Gefitinib in vivo	Jiao [59]
miR-7	• PC-9,RPC-9, H3255, A549 and H1975 cell lines • Xenograft model	Overcomes acquired resistance to EGFR-TKI produced by secondary mutations in EGFR-addicted cancers	MiR-7 reversed resistance to EGFR-TKI in vivo	Rai [52]
miR-7	• A549 cell line	Increased the gefitinib sensitivity through inhibition of IGF1R/PI3K/Akt and EGFR/Raf1/ERK signaling pathways in NSCLC cells	–	Zhao [53]
miR-19a	• 15 serum samples • HCC827, H1975, A549, and PC9 cell lines • Xenograft model	Promoted Gefitinib sensitivity by c-Met targeting in NSCLC cells	MiR-19a decreased Gefitinib resistance in xenograft model	Cao [63]
miR-30a-5p	• H1650, H460, and H1975 cell lines • Xenograft model	Inhibited the PI3K/AKT signaling pathway	MiR-30a-5p increased Gefitinib sensitivity in vivo	Wang [51]
miR-128	• PC9 cell line • Xenograft model	Reversed Gefitinib resistance by reducing the CSC population by inhibiting c-met/PI3K/AKT axis	MiR-128 increased the anti-tumor effect of Gefitinib on NSCLC in xenograft model	Jiang [70]
miR-130a	• A549, H1975, and PC9 cell lines	Promoted Gefitinib sensitivity in NSCLC cells by Met targeting	–	Zhou [58]
miR-133b	• 32 tumor tissue • A549 and H1299 cell lines	Was associated with Erlotinib effectiveness	–	Bisagni [54]
miR-138-5p	• 20 normal and tumor tissues • PC9 and H1975 cell lines	Inhibited angiogenesis by GPR124 down regulation	–	Gao [82]
miR-149-5p	• A549, H1299, H1975 and, PC9 cell lines	LINC00460 sponged miR-149-5p to up regulate IL6 in lung cancer cells	–	Nakano [129]
miR-200a	• H3255, H1975, and HCC827 cell lines	Inhibited NSCLC cells' migration and invasion, while increased Gefitinib sensitivity	–	Zhen [31]
miR-200c	• 150 tumor tissues • PC9, H23, A549, H1975, H460 and H1299 cell lines	Increased Gefitinib sensitivity via ZEB1 targeting	–	Li [110]
miR-200c	• PC9 cell line • Xenograft model	Increased sensitivity to Gefitinib via inhibiting the PI3K/AKT signaling pathway and cell migration by ZEB1 targeting	MiR-200c enhanced sensitivity of tumor cells to Gefitinib and induced apoptosis in xenograft model	Zhou [111]
miR-206	• 37 tumor and 14 normal tissues • PC9 and HCC827 cell lines	Increased Gefitinib sensitivity by blocking the IL6/JAK1/STAT3 pathway in IL6-induced lung cancer resistance cells	–	Yang [127]
miR-206	• HCC827 and PC9 cell lines • Xenograft model	Overcome HGF-induced Gefitinib resistance in EGFR mutant lung cancer cells by AKT/ERK inhibition	MiR-1-3p reversed HGF-induced resistance to Gefitinib in vivo	Jiao [59]
miR-206-3p	• 36 Gefitinib-resistant (GR) and 42 Gefitinib-sensitive (GS) tissues • PC9 cell line • Xenograft model	SNHG14 increased ABCB1 protein expression by miR-206-3p sponging, leading to NSCLC Gefitinib resistance	Silencing of SNHG14 increased sensitivity to Gefitinib in vivo	Wu [169]
miR-223	• PC9 cell line • Xenograft model	Promoted apoptosis in tumor cells by targeting the IGF1R/AKT/S6 signaling pathway and increased Erlotinib sensitivity	Combination of miR-223 and Erlotinib increased sensitivity of tumor cells	Zhao [73]
miR-223	• PC9 cell line • Xenograft model	Inhibited the IGF1R/PI3K/AKT signaling pathway and Erlotinib resistance	MiR-223 reversed resistance to Erlotinib in vivo	Han [157]
miR-365a-5p	• 27 normal and 58 tumor tissues • PC9 cell line • Xenograft model	Reduced cell proliferation and Gefitinib resistance via PELI3 targeting	MiR-365a-5p significantly decreased the tumor size in xenograft model	Li [99]
miR-483-3p	• HCC827, H1975, A549, H292, H1299, and PC9 cell lines • Xenograft model	Promoted Gefitinib sensitivity in NSCLC by decreasing resistant cell growth, inducing apoptosis, inhibition of invasion and migration	Silencing of miR-483-3p promoted Gefitinib resistance in EGFR-mutant NSCLC	Yue [114]
miR-497	• A549 and A549/GR cell lines	Reduced IGF-1R expression and inhibited AKT1 signaling in NSCLC cells	–	Ma [72]
miR-506-3p	• HCC4006 cell line	Under expression of miR-506 activated the SHH pathway and induced EGFR-TKI resistance	–	Haque [172]
miR-506-3p	• 25 normal and tumor tissues • PC9 cell line	Gefitinib resistance via YAP1 regulation in NSCLC cells	-	Zhu [214]
miR-608	• 319 tumor tissues • H1299 and PC9 cell lines	Was prognostic indicator for lung cancer during Gefitinib treatment	–	Zhang [75]
miR-630	• 46 tumor tissues • PC9 and CL97 cell lines	MiR-630 down regulation promoted ERK activation through YAP1 up regulation that resulted in TKI resistance	–	Wu [215]
miR-873	• PC9 cell line	MiR-873 suppression significantly promoted the proliferation of Gefitinib-treated PC9 cells, followed by GLI1 up regulation	–	Jin [175]
miR-1262	• 46 normal and tumor tissues • PC9 and H1299 cell lines	MiR-1262 rs12740674 T allele was correlated with poor prognosis	–	Lei [74]
miR-3127-5p	• 177 normal and tumor tissues • A549 and H292 cell lines • Xenograft model	Inhibited tumor cell growth and invasion by c-Abl targeting	miR-3127-5p decreased NSCLC tumorigenicity and metastasis in xenograft model	Sun [105]
miR-4513	• 319 tumor tissues • H1299 and PC9 cell lines	miR-4513 was prognostic indicator for lung cancer during Gefitinib treatment		Zhang [75]
*Increasing resistance to TKIs*				
miR-21	• 25 plasma samples before treatment • 25 plasma samples after treatment • PC9 cell line • Xenograft model	Inhibited PTEN and PDCD4 expression, while induced PI3K/AKT pathway	Suppression of miR-21 inhibited tumor progression in vivo	Li [143]
miR-23a	• PC9 cell line	miR-23a down regulation increased Erlotinib sensitivity of CSCs through PTEN up regulation	–	Han [144]
miR-26a	• 5 tumor tissues • A549, H520, SW900, H2170, and PC-9 cell lines • Xenograft model	Promoted cell growth and TKI resistance via PTPN13 targeting	miR-26a increased Gefitinib resistance in vivo	Xu [156]
miR-30b	29 plasma samples before treatment 29 plasma samples after treatment	Elevated plasma levels were associated with Erlotinib's inadequate response in EGFR mutant NSCLC patients	–	Hojbjerg [152]
miR-30c	29 plasma samples before treatment 29 plasma samples after treatment	Elevated plasma levels were associated with Erlotinib's inadequate response in EGFR mutant NSCLC patients	–	Hojbjerg [152]
miR-135	• A549, H1650, H1975, H157, and H4006 cell lines	MiR-135 suppression down regulated Bcl-2 while up regulated Bax that resulted in increased apoptosis	–	Wang [130]
miR-135a	• NCI-H1650 and • NCl-H1975 cell lines	Stimulated cell proliferation, invasion, and Gefitinib resistance in NSCLC cells through RAC1 targeting	–	Zhang [160]
miR-200c	• HCC4006 cell line	MiR-200c/LIN28B axis plays an essential role in acquired EGFR-TKI resistance	–	Sato [113]
miR-214	• 7 EGFR mutant tumor tissues • HCC827 cell line	Promoted Erlotinib resistance through LHX6 targeting	–	Liao [191]
miR-214	• HCC827 cell line	Gefitinib resistance in EGFR mutant cell lines was achieved through interplay with the PTEN/AKT signaling pathway	–	Wang [142]
miR-223	• HCC827 cell line • Xenograft model	Inhibited the IGF1R/PI3K/AKT signaling pathway and Erlotinib resistance	miR-223 reversed the resistance to Erlotinib in xenograft model	Han [157]
miR-223	• HCC827 cell line	Promoted the AKT and Notch signaling pathways and modulated NSCLC cell susceptibility to Erlotinib by regulating FBXW7	–	Zhang [224]
miR-299-3p	• 44 sensitive tumor tissues • 40 resistant tumor tissues • A549, H1975, H1299, • HCC827 and PC9 cell lines • Xenograft model	RHPN1-AS1 regulated Gefitinib resistance in NSCLC by miR-299 targeting	Combination of RHPN1-AS1 and Gefitinib inhibited tumor growth in vivo	Li [205]
miR-451	• A549 and H1975 cell lines • Xenograft model	NOTCH-1 promoted Gefitinib resistance in LUAD cells through SNHG15/miR-451/ZEB1 axis in vivo and in vitro	SNHG15/miR-451/ZEB1 axis induced Gefitinib resistance in xenograft model	Huang [223]
miR-641	• 18 T tissues • 18 resistant T tissues • PC9 cell line • Xenograft model	Promoted Erlotinib resistance in NSCLC cells by NF1 targeting	Combination of miR-641 and Erlotinib increased apoptosis and decreased tumor cells proliferation in vivo	Chen [94]
miR-762	• 59 tumor tissues • A549, NCI-H820, NCI-H2170, NCI-H1650, NCI-H1993, NCI-H2126, NCI-H1975, NCIH1299, NCI-H1648, NCI-H1703, NCI-H2347 and PC9 cell lines • Xenograft model	Gefitinib resistance in NSCLC cells by mediating the IL6/STAT3 pathway	miR-762 promoted Gefitinib resistance and increased tumor formation in a xenograft model	Ge [128]

## Receptor tyrosine kinases (RTKs)

RTKs are trans-membrane proteins that are expressed on different cell types and are implicated in a myriad of cellular mechanisms including proliferation, cell survival, and cell–cell communication [35]. Deregulation of RTKs leads to the development of different diseases, most particularly, malignancies as it is reported that approximately 30% of RTKs are mutated or aberrantly expressed in different human cancers [36]. EGFR is a trans-membrane glycoprotein with an intracellular tyrosine kinase domain that is autophosphorylated to activate MAPK and PI3K pathways [3, 37–40]. EGF triggers the tyrosine kinase activity of EGFR that regulates cell proliferation, migration, and apoptosis [41]. There is EGFR up regulation in about 60% of NSCLC patients [42]. In NSCLC patients with activating EGFR mutations, EGFR tyrosine kinase inhibitors (EGFR-TKI) have a remarkable impact and prolonged survival compared with standard treatments [43–45]. Gefitinib as an EGFR-TKI has been authorized for patients with EGFR mutations in exon 19 (deletions) or exon 21 (Leu858Arg) [46, 47]. Compared with platinum-based combination chemotherapy, Gefitinib delays tumor progression and enhances overall survival. Nevertheless, many individuals develop resistance to TKIs throughout treatment [40, 48, 49]. Due to an EGFR T790M mutation in exon 20, around 50% of patients who initially responded to EGFR-TKI developed resistance to EGFR-TKI [50]. EGFR and IGF1R suppression can inhibit the PI3K/AKT signaling pathway. It has been shown that Gefitinib and *miR-30a-5p* mimics reduced EGFR-TKIs resistance [51]. The delivery of miR-7 plasmids through cationic liposomes may be exploited to overcome acquired resistance to EGFR-TKI produced by secondary EGFR mutations [52]. *MiR-7* increased the Gefitinib sensitivity through suppression of IGF1R and EGFR signaling pathways in NSCLC cells [53]. It has been shown that up regulation of *miR-133b* and *miR-146a* while *miR-7* down regulation was associated with Erlotinib effectiveness in NSCLC. Since, there was a correlation between *miR-133b* up-regulation and prolonged Progression-Free Survival (PFS) in NSCLC patients taking Erlotinib, it is postulated that *miR-133b* might improve Erlotinib sensitivity. MiR-200c also increased Gefitinib sensitivity [54]. *APCDD1L-AS1* promoted Icotinib resistance through miR-1322/miR-1972/miR-324-3p sponging that up regulated the SIRT5 and EGFR in lung tumor cells. SIRT5 inhibited the EGFR autophagic degradation to induce Icotinib resistance [55].

Met is a RTK that can be activated by Hepatocyte Growth Factor (HGF) to promote the MAPK and PI3K/AKT downstream pathways. It is involved in EGFR-TKI resistance of NSCLC patients [56, 57]. There was *miR-130a* up regulation in Gefitinib-sensitive NSCLC cells that promoted Gefitinib sensitivity in NSCLC cells by Met targeting [58]. *MiR-1-3p* and *miR-206* may also overcome HGF-induced Gefitinib resistance via suppression of c-Met signaling in EGFR mutant lung cancer cells [59]. Tumor Immune Microenvironment (TIME) alterations have been examined before and after the development of EGFR-TKI resistance. It has been found that *miR-1* increased EGFR-TKI sensitivity by reduction of the CD8 + T cells migration. *MiR-1* inhibited monocytes and lymphocytes motility by cytokines down regulations. *MiR-1* reduced intratumoral CD8 + T cells in EGFR-TKI resistant lung cancer patients [60]. EGFR has also been shown to interact with the c-Met regularly [61, 62]. It has been shown that *miR-200a* reduced cell invasion and Gefitinib resistance in NSCLC cells through EGFR and c-Met down regulations [31]. There were also significant reduced serum *miR-19a* levels in Gefitinib resistant patients. *MiR-19a* down regulation promoted Gefitinib resistance and Epithelial-to-Mesenchymal Transition (EMT) in Gefitinib-sensitive NSCLC cells. *MiR-19a* promoted Gefitinib sensitivity by c-Met targeting in NSCLC cells [63]. Cancer Stem Cells (CSCs) are a group of tumor cells involved in chemo resistance [64]. Since, CSCs can differentiate and cause diverse cell populations to form tumor bulks, they are considered as the main tumor-initiating cells [65]. CD133 + populations of NSCLC-CSCs are responsible for increased chemotherapeutic resistance and tumor relapse [66, 67]. HGF/c-Met signaling induced tumor progression through PI3K/AKT pathway [68, 69]. There was *miR-128* down regulation in PC9-CSCs. *MiR-128* reversed Gefitinib resistance via c-Met/PI3K/AKT inhibition and reducing the CSC population [70].

Insulin-like Growth Factor-1 Receptor (IGF1R) belongs to RTKs protein family that can be activated by autophosphorylation in conjunction with the Insulin-like Growth Factors (IGFs) to promote MAPK and PI3K/AKT signaling pathways. Therefore, IGF1R can control cell proliferation, differentiation, metabolism, and apoptosis [71]. It is hypothesized that aberrant IGF1R activation may increase the PI3K/AKT signaling pathway, thus conferring resistance to EGFR-TKIs. It has been shown that *miR-497* can also regulate NSCLC's resistance to Gefitinib. *MiR-497* reduced IGF1R expression and inhibited AKT1 signaling in NSCLC cells. *MiR-497* may influence tumor cell responsiveness to chemotherapy and tumor cell resistance to EGFR-TKI via IGF1R targeting and AKT activation [72]. It has also been reported that *miR-223* promoted apoptosis in tumor cells by targeting the IGF1R/Akt/S6 signaling pathway and increased Erlotinib sensitivity [73].

The prognostic impact of the *miR-1262* rs12740674 variation has been investigated in advanced lung cancer patients treated by EGFR-TKIs. The rs12740674 T allele was correlated with a worse prognosis. The *miR-1262* rs12740674 T allele was substantially related to poor prognosis following EGFR-TKI treatment. *MiR-1262* also significantly increased the susceptibility of lung adenocarcinoma cells to Gefitinib [74]. *MiR-4513* rs2168518 and *miR-608* rs4919510 polymorphisms were also substantially correlated with the prognosis of lung cancer patients treated with Gefitinib. Carriers of homozygous CC variant of rs4919510 had significantly longer OS and PFS than those with the GG variant. The carriers of heterozygous GA variant of rs2168518 had also a better prognosis in comparison with the GG variant. The rs4919510 and rs2168518 polymorphisms were prognostic indicators for lung cancer during Gefitinib treatment [75].

G-protein Coupled Receptor 56 (GPR56) has a pivotal role in cell adhesion and angiogenesis [76]. GPR124 functions as an angiogenesis regulator, and abnormal tumor angiogenesis have been associated with anti-EGFR therapeutic resistance [77–81]. Gefitinib-resistant cell lines were sensitized to Gefitinib using *miR-138-5p*. Gefitinib resistance was associated with *miR-138-5p* down regulation. Elevated amounts of *miRNA-138-5p* down-regulated GPR124 in PC9 cells, and this modulation contributed to drug sensitivity [82].

## MEK/ERK and JAK/STAT signaling pathways

The Mitogen-Activated Protein Kinase (MAPK) pathway regulates diverse cellular mechanisms including proliferation, differentiation, and motility [83]. MAPK pathway is activated by kinase cascades. Consecutive activation of the MAPK Kinase Kinase (MAPKKK) and MAPK Kinase (MAPKK) promote a specific MAPK which then phosphorylates different proteins in the cytosol and nucleus to exert its biological impacts through alterations in protein function and gene transcription [84]. Three main subfamilies of MAPKs include ERK, JNK, and p38 [85]. Compromised MAPK signaling has been correlated with diabetes, cancers, and neurodegenerative disorders [86, 87]. Increased p38-MAPK pathway activity was demonstrated to be associated with higher MDR1 expression and multidrug resistance in tumor cells [88]. The MEK/ERK pathway is a crucial downstream signaling cascade required for cell growth and neoplastic transformation. As a result, MEK inhibitors have been intensively explored to treat different solid tumors in clinical trials [89, 90]. MEK-inhibitors have clinical responses in some patients, however a minority of tumors are resistant [91, 92]. It has been reported that up-regulation of *miR-17-92* via activation of the STAT3 pathway caused MEK inhibitor resistance. Concurrent suppression of the MEK and STAT or *miR-17* sensitized resistant cells to AZD6244 treatment substantially via Bcl-2 Interacting Protein (BIM) up-regulating [93]. Neurofibromin 1 (NF1) is a GTPase-activating protein that inhibits the Ras signaling pathway, which, in turn, inhibits the MAP-ERK kinase *MiR-641* up-regulation in EGFR-TKI-resistant NSCLC cells promoted Erlotinib resistance in NSCLC cells by directly targeting NF1 via activation of ERK signaling. *MiR-641* may also render EGFR-TKI-resistant NSCLC cells susceptible to TKI therapy [94]. PELI3 is a scaffolding protein that promotes the ETS Like-1 protein (Elk-1) and c-Jun signaling pathways and regulates innate immune responses [95, 96]. PELI3 is an E3 ubiquitin protein ligase with a function in insulin resistance and inflammation [97]. Elk1 needs to be phosphorylated by MAPKs to activate the FOS proto-oncogene [98]. There was PELI3 up-regulation in NSCLC cell lines and tissues which was associated with a poor prognosis. *MiR-365a-5p* reduced cell proliferation and Gefitinib resistance via Pellino E3 Ubiquitin Protein Ligase Family Member 3 (PELI3) targeting [99].

Abelson Tyrosine-Protein Kinase 1 (ABL1) is a cytoplasmic and nuclear tyrosine kinase involved in cell proliferation, adhesion, and stress response [100]. The c-Abl promotes cell proliferation and tumorigenesis in various cancers [101–103]. Activated c-Abl also phosphorylates the EGFR that is resulting in reduced EGFR internalization and increased EGFR expression. The c-Abl directly interacts with the Grb2 to activate Ras/ERK pathway [104]. There was significant *miR-3127-5p* down regulation in recurrent NSCLC tumor tissue compared with initial tumors. *MiR-3127-5p* expression was significantly correlated with advanced tumor stage in NSCLC. *MiR-3127-5p* significantly reduced tumor cell growth and invasion by targeting the c-Abl and regulating the c-Abl/Ras/ERK pathway. Dasatinib sensitivity was also associated with *miR-3127-5p* down regulation in NSCLC cells [105].

EMT is characterized by Cadherin 1 (CDH1) down-regulation and up regulation of the mesenchymal biomarkers. EMT increases tumor cell motility, invasion, and drug resistance. Acquiring the mesenchymal phenotype is linked with chemo resistance and confers primary resistance to Trastuzumab, a HER2/neu inhibitor [106, 107]. EMT has been related to resistance to EGFR-TKIs in NSCLC patients. Mesenchymal markers are also expressed in clinical samples with EGFR-TKI resistance [108, 109]. Zinc finger E-box-binding homeobox (ZEB) family proteins as the CDH1 transcriptional repressors are pivotal targets in the miRNA-mediated EMT process. It has been shown that *miR-200c* regulated EMT and increased Gefitinib sensitivity via ZEB1 targeting in NSCLC cells. Patients with *miR-200c* up-regulation may benefit more from EGFR-TKIs compared with *miR-200c* down-regulation. *MiR-200c* inhibited MEK/ERK pathway to re-sensitize Gefitinib resistant NSCLC cells [110]. *MiR-200c* increased drug-resistant PC9-ZD sensitivity to Gefitinib via ZEB1 targeting [111]. LIN28B and LIN28A are RNA-binding proteins that have many biological roles. LIN28 family reprograms the somatic cells to pluripotent stem cells in combination with self-renewal transcription factors [112]. The miR-200c/LIN28B axis is required to maintain EGFR-TKI resistance cells with EMT features. This axis has an essential role in EGFR-TKI resistance [113]. There was significant *miR-483-3p* down regulation in Gefitinib-resistant NSCLC cells and lung tissues. *MiR-483-3p* promoted Gefitinib sensitivity in NSCLC by decreasing resistant cell growth and inducing apoptosis. It also decreased EMT phenotype and metastasis in Gefitinib-resistant NSCLC cells. Moreover, *miR-483-3p* down regulation activated the FAK/ERK pathway through up-regulating integrin β3. The *miR-483-3p* suppression in Gefitinib-resistance cells was related to promoter hyper methylation [114].

The JAK/STAT signaling pathway is implicated in multiple pathophysiological mechanisms such as cell proliferation, differentiation, immunity, cytokine functions, and tumorigenesis [115, 116]. Following the interaction of cytokines with the receptor, JAKs phosphorylate the STATs to form a dimer that translocates into the nucleus to induce the transcription of target genes [117]. Higher STAT3 activation is associated with increased risk of recurrence and shorter survival in different cancers [118]. JAK/STAT pathway also leads to the failure of conventional chemotherapy via promoting the expression of EMT-inducing transcription factors [119]. STAT3 is an oncogenic transcription factor regularly activated in cancer and tumor-related myeloid cells by the IL-6 [120]. IL6-induced STAT3 deregulation is associated with tumor progression in various cancers [121–123]. IL6/STAT3 signaling may result in drug resistance [124–126]. EGFR mutant lung cancer cells evade Gefitinib therapy by over-activating STAT3 via miR-206 down regulation [127]. ABR regulates various biological activities by inhibiting the small GTPase Rac activity. ABR dysfunction is associated with IL-6 activation. Hypoxia stimulates the GTP-bound form of Rac, resulting in increased IL-6 production during the pathogenesis of pulmonary hypertension. There was significant *miR-762* up regulation in Gefitinib-resistant NSCLC cells compared to parental cells. Increased expression of *miR-762*, which is mediated by the IL6/STAT3 signaling pathway, resulted in Gefitinib resistance in NSCLC cells [128]. *LINC00460* is a competitive endogenous RNA decoy for the *miR-149-5p* that consequently boosts IL-6 production and EMT-like characteristics in lung cancer cells. Patients with a high *LINC00460* expression had a substantially lower PFS and OS after Gefitinib treatment [129]. It has been reported that *miR-135* promoted Gefitinib resistance in NSCLC cells. *MiR-135* down-regulated CDH1 and b-catenin while up-regulated PD-L1. *MiR-135* inhibition affected the NSCLC cells by TRIM16 up-regulation. JAK/STAT signaling pathway was also implicated in *miR-135* and TRIM16 regulation. *MiR-135* suppression down-regulated Bcl-2 while up-regulated Bax that increased apoptosis [130].

## PI3K/AKT signaling pathway

PI3K/AKT pathway is an intracellular signal transduction pathway that plays a crucial role in regulating cellular metabolism, proliferation, growth, and angiogenesis in response to extracellular signals [131, 132]. Following activation of PI3K by growth factors [133, 134], AKT is phosphorylated, activated, and localized in the plasma membrane and can exert different downstream effects such as CREB and mTOR activation, p27 inhibition, and FOXO localization in the cytoplasm [134–136]. The abnormal PI3K/AKT activation in cancer cells promotes the expression of ATP-Binding Cassette (ABC) transporters, inhibits apoptosis, and induces tumor growth, thereby contributing to the reduced response to chemotherapeutic medications [137]. EGFR-TKIs might reduce EGFR downstream pathway activity, primarily through the PI3K/AKT pathway, which inhibits cell proliferation, invasion, and induction of apoptosis [138]. Phosphatase and Tensin Homolog (PTEN) as a suppressor of PI3K/AKT pathway is associated with EGFR-TKIs resistance [139, 140]. PTEN is a tumor suppressor protein that converts PIP3 to PIP2 to inhibit the PI3K/AKT pathway [141]. There was a significant up-regulation of *miR-214* in HCC827/GR. *MiR-214* regulated the PTEN/AKT signaling pathway in NSCLC EGFR mutant cells. The potential of *miR-214* to modulate acquired resistance to Gefitinib in EGFR mutant cell lines was achieved through interplay with the PTEN/AKT signaling pathway [142]. There was *miR-21* up regulation in advanced EGFR-TKI resistant NSCLC patients. *MiR-21* promoted EGFR-TKI resistance via PTEN and PDCD4 targeting that resulted in PI3K/AKT induction [143]. There was significant *miR-23a* up-regulation in CD133 positive PC9 CSCs. Inhibition of *miR-23a* increased Erlotinib sensitivity of CSCs through PTEN up regulation [144].

IGF1R is involved in neoplastic transformation and drug resistance of a wide variety of tumors [145, 146]. The PI3K/AKT pathway as one of the common EGFR downstream signaling pathways are activated by IGF1R [147]. IGF1R activity is related to EGFR-TKI resistance in NSCLC cell lines and lung cancer patients [148, 149]. IGF1R-TKI can overcome EGFR-TKI resistance in vitro and in vivo [147, 150]. It has been shown that *miR-30a-5p* down regulated PIK3R2, hence lowering the amount of p-AKT in cell lines. *MiR-30a-5p* inhibited cell migration while promoted apoptosis by PIK3R2 targeting. Therefore, *miR-30a-5p* in combination with other EGFR-TKI agents increased tumor cell drug sensitivity [151]. Elevated plasma levels of *miR-30b* and *miR-30c* were also associated with Erlotinib's inadequate response in EGFR mutant NSCLC patients [152].

Protein Tyrosine Phosphatase Non-Receptor Type 13 (PTPN13) belongs to the non-receptor tyrosine phosphatases that functions as a tumor suppressor in NSCLC [153]. PTPN13 dephosphorylates oncogenic proteins including TRIP6, HER2, and Insulin Receptor Substrate 1 (IRS-1) [153, 154]. It can also regulate the PI3K signaling via PIK3R2 dephosphorylation [155]. There was significant *miR-26a* up regulation in TKI-resistant NSCLC cells that promoted cell growth and TKI resistance via PTPN13 targeting. *MiR-26a* also activated Src by PTPN13 targeting that promoted EGFR signaling [156]. PI3K inhibitors significantly increase NSCLC cell susceptibility to drug-induced apoptosis. It has been shown that *miR-223* stimulated the IGF1R/PI3K/AKT signaling pathway and Erlotinib resistance in PC9/CD133 + cells [157]. Ras-related C3 botulinum toxin substrate 1 (Rac1) is a small GTPase from the Rho protein family that belongs to the Ras superfamily [158]. Rac1 is involved in various activities in cell differentiation, migration, proliferation, vesicle trafficking, and cytoskeletal dynamics [158, 159]. It has been reported that *miR-135a* stimulated cell proliferation, invasion, and Gefitinib resistance in NSCLC cells via Rac1 targeting and regulation of PI3K/AKT pathway [160].

## Sonic hedgehog (SHH), wingless/int (WNT), and nuclear factor-kB (NF-kb) signaling pathways

The Hedgehog (Hh) pathway is a highly conserved signal transduction pathway that functions in cellular communications during embryonic development and is implicated in organogenesis, homeostasis, and regeneration [161]. Studies have indicated that aberrant activation of the Hh pathway induces cell proliferation and differentiation which culminates in tumorigenesis [162, 163]. Hyper-activation of Hh pathways is frequently observed in esophageal cancers [164], and it also promotes prostate cancer progression [165]. Gli-1 up-regulation was observed in residual esophageal tumors after chemo-radiotherapy [166]. Hh signaling confers multidrug resistance through regulating the expression of ABC transporters family including Multidrug Resistance Protein 1 (MDR1) [167]. Drug efflux through the ABC transporters is considered the most important mechanisms of Multidrug Resistance (MDR) [168]. There was Small Nucleolar RNA Host Gene 14 (*SNHG14*) up-regulation in Gefitinib-resistant NSCLC tissues and cells. *SNHG14* increased ABCB1 protein expression by *miR-206-3p* sponging, leading to NSCLC Gefitinib resistance [169]. MiRNAs have a pivotal role in the regulation of the Sonic Hedgehog (SHH) pathway, which is essential in organogenesis and embryogenesis [170, 171]. It has been reported that under expression of *miR-506* activated the SHH pathway promoted EGFR-TKI, migration, and EMT process. The expression of miR-506-3p was significantly reduced in Erlotinib-resistant cells [172]. GLI1 is the main transcription factor of Hh signaling pathway [173, 174]. It has been observed that *miR-873* suppression significantly induced the proliferation of Gefitinib-treated PC9 cells, followed by GLI1 up-regulation. *MiR-873* suppression also enhanced angiogenesis and Gefitinib resistance in NSCLC cells [175].

WNT family is a group of secreted glycoproteins that functions as ligands binding to a Frizzled (Fz) family cell surface receptor to activate downstream signaling cascades [176, 177]. RTK and LRP-5/6 serve as co-receptors to facilitate the WNT ligand and Fz receptor interaction [178]. The signals are then transduced via canonical Wnt/β-catenin, non-canonical Wnt/calcium, and non-canonical Planar Cell Polarity (PCP) pathways [179]. Wnt pathway is involved in cellular differentiation, proliferation, maturation, and tumorigenesis [180–182]. Wnt signaling is involved in progression of various cancers including hepatocellular carcinoma, prostate, ovarian, pancreas, and breast cancers [183–187]. The Wnt pathway is responsible for the resistance of cancer cells to traditional chemotherapy and radiotherapy through regulating stemness and maintaining the CSCs [176]. LIM Homeobox (LHX) as the main subfamily of homeobox genes are involved in various malignancies [188]. LHX6 inhibits the Wnt/b-catenin signaling pathway to suppress the breast cancer cells proliferation and invasion [189]. It has a critical role in lung cancer via regulation apoptosis and cell cycle-related genes such as Tumor protein P53 (p53), B-Cell Lymphoma 2 (BCL-2), Cyclin D1 (CCND1), and Cyclin Dependent Kinase Inhibitor 1A (CDKN1A) [190]. There was *miR-214* up regulation in the plasma of NSCLC patients who acquire EGFR-TKI resistance. *MiR-214* promoted the Erlotinib resistance and the metastasize potential in HCC827 cells through LHX6 targeting [191].

The NF-kB encompasses a family of closely related transcription factors implicated in cell survival, immune responses, and cytokine production [192, 193]. The NF-kB activation is achieved through the non-canonical and canonical pathways [194]. In the canonical pathway, NF-kB is activated following targeted phosphorylation and subsequent degradation of IkB [195]. NF-kB signaling is involved in inflammatory and autoimmune disorders as well as cancers [194, 196, 197]. It has also been reported that NF-kB exerts an anti-apoptotic role and promotes drug resistance in tumor cells [198–200]. In contrast, inhibition of NF-kB enhances cancer cell sensitivity to chemotherapeutic drugs via MDR1 down regulation [201]. Interleukin 1 Receptor Associated Kinase 1 (IRAK1) is serine/threonine kinase implicated in the NF-kB-regulated inflammatory response, antiapoptosis, and tumor development [202]. It has been reported that there was significant *miR-146b-5p* down regulation in EGFR TKI-resistant cells. *MiR-146b-5p* increased EGFR TKIs sensitivity by targeting IRAK1 [203].

TNF Superfamily Member 12 (TNFSF12) belongs to the Tumor Necrosis Factor (TNF) protein family expressed in a range of organs, immune cell types, and tumor cells, which activates caspase 8 and caspase 9 and cause extrinsic and intrinsic apoptosis cascades [204]. *RHPN1-AS1* was down-regulated in Gefitinib-resistant NSCLC patients and cell lines. *RHPN1-AS1* regulated Gefitinib resistance in NSCLC by targeting the *miR-299-3p/TNFSF12* pathway. *TNFSF12* also induced apoptosis in Gefitinib resistant tumor cells [205].

## Hippo and NOTCH signaling pathways

The Hippo signaling is a structurally and functionally conserved pathway that has pivotal functions in regulation of organ size, cell proliferation, apoptosis, and tissue regeneration [206, 207]. Following the activation of the Hippo pathway, Mammalian Sterile 20-like kinase (MST1/2) is phosphorylated and promotes the activation of Large Tumor Suppressor (LATS1/2), which controls gene expression through phosphorylating and inhibiting the activity of the transcriptional co-activator proteins Yes-Associated Protein (YAP) and transcriptional co-activator with PDZ-binding motif (TAZ) [208, 209]. The Hippo pathway exerts tumor-suppressive functions and its mutations lead to the overgrowth of the affected cells [210]. Deregulation of the Hippo pathway also renders cancer cells resistant to chemotherapy [211]. Downregulation of MST1 levels has been correlated to cisplatin resistance in prostate tumor cells [212]. On the other hand, YAP up-regulation was associated with resistance to taxane-based therapy in ovarian cancer [213]. There was *miR-506-3p* down regulation in NSCLC cells. *MiR-506-3p* reduced cell viability and apoptosis in PC-9GR cells after Gefitinib therapy. *MiR-506-3p* enhanced Gefitinib-induced Bcl-2 down regulation and Bax up regulation in PC 9GR cells. *MiR-506-3p* down regulation was associated with Gefitinib resistance via YAP1 regulation in NSCLC cells [214]. It has been shown that *miR-630* down regulation may predict an adverse response to TKI treatment and a poor prognosis in lung adenocarcinoma. TKI resistance in EGFR-mutated lung cancer cells may be due to a feedback loop between miR-630-YAP1-ERK due to a Bad down-regulation caused by ERK signaling-induced phosphorylation. *MiR-630* down regulation promoted ERK activation through YAP1 up regulation that resulted in TKI resistance. The miR-630/YAP1/ERK axis promotes TKI resistance in EGFR-mutated lung tumor cells [215].

Notch signaling is a cell–cell communication pathway involved in multiple cellular processes including embryonic development, proliferation, differentiation, EMT, migration, and apoptosis [216]. Following the interaction of the Notch receptors (NOTCH1-4) with a ligand–protein such as Delta-Like (DLL) and Jagged, proteolytic cleavage is induced and the intracellular domain is released which enters the nucleus to regulate the transcription of target genes [217, 218]. Aberrancies in this pathway have been correlated with a variety of developmental disorders of the heart, kidney, liver, and skeleton as well as malignancies [219, 220]. Moreover, Notch signaling is associated with drug resistance by promoting the formation of CSCs and mediating the EMT process [221, 222]. There were reduced levels of *SNHG15* expression in Gefitinib-resistant LUAD cells due to NOTCH1 impairment. In Gefitinib-resistant cells, lack of *SNHG15* inhibited cell proliferation, migration, and EMT processes while promoting cell death. NOTCH-1 promoted Gefitinib resistance through SNHG15/miR-451/ZEB1 axis [223]. There was *miR-223* up-regulation in the Erlotinib-resistant HCC827 cells compared with parental. Increased expression of *miR-223* stimulated the AKT and Notch signaling pathways in Erlotinib-resistant cells. *MiR-223* may be a critical onco-miRNA that modulates NSCLC cell susceptibility to Erlotinib by regulating FBXW7. Erlotinib-resistant NSCLC patients may benefit from a new therapy that targets the Notch/miR-223/FBXW7 pathway [224].

## Conclusions

TKIs are effective therapeutic modalities in the targeted therapy of various cancers; however they cause various side effects in cancer patients. Therefore, it is required to detect the lung cancer patients who are resistant toward the TKIs to manage the therapeutic methods and reduce side effects. Circulating miRNAs are tolerant toward different pH conditions and ambient temperature, showing their importance as efficient diagnostic and prognostic tumor markers. Since, miRNAs are involved in response to the anti-cancer drugs, drug response can be predicted by the miRNAs expression profiling. In the present review we have summarized specific miRNAs involved in the regulation of TKIs responses in lung tumor cells. It was observed that miRNAs affect the TKIs via regulation of various signaling pathways including NOTCH, WNT, PI3K/AKT, Hippo, and JAK/STAT. All of these signaling pathways finally regulate the EMT through the specific transcription factors such as YAP, GLI1, ZEB1, and CSL. This review clarifies the molecular interactions between the miRNAs and signaling pathways during the TKIs response in lung tumor cells that paves the way of introducing a miRNA-based panel marker for detection of TKIs response in lung cancer patients. However, there is still a lack of miRNAs serum sample assessment in the majority of discussed reports. Therefore, in future studies it is necessary to evaluate the levels of miRNAs expressions in serum samples of lung cancer patients to introduce such factors as the non-invasive markers of TKIs response prediction.

## Data Availability

Not applicable.

## Abbreviations

ABC: ATP-binding cassette
ABL1: Abelson tyrosine-protein kinase 1
AKT: Protein kinase B
BCL-2: B-cell lymphoma 2
BIM: Bcl-2 interacting protein
CCND1: Cyclin D1
CDH1: Cadherin 1
CDKN1A: Cyclin dependent kinase inhibitor 1A
CSCs: Cancer stem cells
DLL: Delta-like
EGFR-TKI: EGFR tyrosine kinase inhibitors
EGFR: Epidermal growth factor receptor
EMT: Epithelial-to-mesenchymal transition
Elk-1: ETS like-1 protein
Fz: Frizzled
GPR56: G-protein coupled receptor 56
HGF: Hepatocyte growth factor
Hh: Hedgehog
IGF1R: Insulin-like growth factor-1 receptor
IGFs: Insulin-like growth factors
IRAK1: Interleukin 1 receptor associated kinase 1
IRS-1: Insulin receptor substrate 1
JAK: Janus kinase
LATS1/2: Large tumor suppressor
LHX: LIM homeobox
MAPK: Mitogen-activated protein kinase
MAPKK: MAPK kinase
MAPKKK: MAPK kinase kinase
MDR1: Multidrug resistance protein 1
MDR: Multidrug resistance
MST1/2: Mammalian sterile 20-like kinase
MiRNAs: MicroRNAs
NF-kB: Nuclear factor-kB
NF1: Neurofibromin 1
NRTKs: Non-receptor tyrosine kinases
NSCLC: Non-small-cell lung cancer
PCP: Planar cell polarity
PELI3: Pellino E3 ubiquitin protein ligase family member 3
PFS: Progression-free survival
PI3K: Phosphatidylinositol 3-kinase
PTEN: Phosphatase and tensin homolog
PTPN13: Protein tyrosine phosphatase non-receptor type 13
p53: Tumor protein P53
RTKs: Receptor tyrosine kinases
Rac1: Ras-related C3 botulinum toxin substrate 1
SCLC: Small-cell lung cancer
SHH: Sonic hedgehog
SNHG14: Small nucleolar RNA host gene 14
STAT: Signal transducer and activator of transcription
TAZ: Transcriptional co-activator with PDZ-binding motif
TIME: Tumor immune microenvironment
TKIs: Tyrosine kinase inhibitors
TKRs: Tyrosine kinase receptors
TNF: Tumor necrosis factor
TNFSF12: TNF superfamily member 12
WNT: Wingless/int
YAP: Yes-associated protein
ZEB: Zinc finger E-box-binding homeobox
